# Predicting health related deprivation using loyalty card digital footprints

**DOI:** 10.23889/ijpds.v8i3.2282

**Published:** 2023-09-11

**Authors:** Gavin Long, Georgiana Nica-Avram, John Harvey, Roberto Mansilla, Simon Welham, Evgeniya Lukinova, James Goulding

**Affiliations:** 1 N/LAB, NUBS, University of Nottingham; 2 School of Biosciences, The University of Nottingham, Nottingham, UK

## Introduction & Background

In England, The Indices of Deprivation (IoD) are a widely used and referenced measure to assess local levels of deprivation across a range of domains, including health and disability. However, due to their complex nature and the number of inputs required to generate these measures, they are only updated infrequently. Typically every 4-5 years, with the most recent versions released in 2019 and 2015.

This study expands on previous research looking at the feasibility of using digital footprint data, in the form of retail loyalty card transactions, to predict local deprivation. This work focuses specifically on the health and disability subdomain of IoD. Our hypothesis is that retail behaviour relating to food purchases and their associated nutritional content, can be used to predict health deprivation.

## Objectives & Approach

The work utilises loyalty card data from a large UK grocery retailer. Anonymised geo-location data for loyalty card members was used to assign retail grocery transactions to individual Lower Layer Super Output Areas (LSOAs) for each of the ten quarters in the study period (July 2019 - December 2021). A nutritional lookup was developed to enable the nutritional content of food transactions to be assigned to each LSOA.

A number of metrics based on categories of food sold and their nutritional content were developed and used in a Machine Learning model, based on a Random Forest classifier, to predict areas with high levels of health related deprivation.

## Relevance to Digital Footprints

This study uses data derived from digital footprint data of grocery transactions. It demonstrates the potential for utilising digital footprint data as a proxy for traditional demographic data without the need for expensive, both in terms of time and cost, surveying to be performed.

## Results

The random forest classifier was able to predict neighbourhoods (at the LSOA level) with the top 20% of health related deprivation. A high level of predictive power was identified (Overall accuracy 80%). SHAP (SHapley Additive exPlanations) and Model Class Reliance (MCR) were used to determine the importance of the input features. Areas with higher proportional spending on cigarettes and soft drinks and lower spending on fish, wine and fruit and vegtables were found to be associated with extreme levels of health deprivation. In terms of nutrition, two derived metrics, calories per pound spend and the obesogenicity of food purchased, were found to be important predictors of health deprivation.

## Conclusions & Implications

Digital footprint data on grocery purchases have been shown to be highly effective at predicting areas of extreme health related deprivation at the LSOA level. Features related to proportional spend on food categories and proportions of nutrients associated with these purchases were identified as optimal for predicting health related deprivation. 

The number of calories per pound spent and, to a lesser extent, the proportion spent on cigarettes, in an LSOA was found to be the most important predictor of high levels of health related deprivation.

The high level of predictive accuracy obtained offers the potential for using digital footprint data as a proxy for traditional deprivation measures. This could enable rapid and near real-time surveillance of areas with poor health outcomes compared to traditional approaches. This could allow early interventions to be put in place mitigating some of the negative impacts of health related deprivation.

